# Highly Sensitive Chemiluminescence-Based Lateral Flow Immunoassay for Cardiac Troponin I Detection in Human Serum

**DOI:** 10.3390/s20092593

**Published:** 2020-05-02

**Authors:** Gyeo-Re Han, Min-Gon Kim

**Affiliations:** Department of Chemistry, Gwangju Institute of Science and Technology (GIST), 123 Cheomdangwagi-ro, Buk-gu, Gwangju 61005, Korea; gyore@gist.ac.kr

**Keywords:** enzyme-based biosensor, chemiluminescence immunoassay, lateral flow assay, high-sensitivity, cardiac troponin I

## Abstract

Lateral flow assays (LFAs) have become the most common biosensing platforms for point-of-care testing due to their compliance with the ASSURED (affordable, sensitive, specific, user-friendly, rapid/robust, equipment-free, and deliverable to end-users) guidelines stipulated by the World Health Organization. However, the limited analytical sensitivity and low quantitative capability of conventional LFAs, which use gold nanoparticles (AuNPs) for colorimetric labeling, have prevented high-performance testing. Here, we report the development of a highly sensitive chemiluminescence (CL)-based LFA involving AuNPs conjugated with aldehyde-activated peroxidase and antibody molecules—i.e., AuNP-(ald)HRP-Ab—as a new conjugation scheme for high-performance testing in LFAs. When paired with the CL-based signal readout modality, the AuNP-(ald)HRP-Ab conjugate resulted in 110-fold enhanced sensitivity over the colorimetric response of a typical AuNP-Ab conjugate. To evaluate the performance of the CL-based LFA, we tested it with human cardiac troponin I (cTnI; a standard cardiac biomarker used to diagnose myocardial infarction) in standard and clinical serum samples. Testing the standard samples revealed a detection limit of 5.6 pg·mL^−1^ and acceptably reliable precision (with a coefficient of variation of 2.3%–8.4%), according to clinical guidelines. Moreover, testing the clinical samples revealed a high correlation (r = 0.97) with standard biochemical analyzers, demonstrating the potential clinical utility of the CL-based LFA for high-performance cTnI testing.

## 1. Introduction

Cardiac troponin proteins control the contractile activity of cardiac muscles by regulating calcium-mediated interactions between actin and myosin [1]. After cardiac muscle undergoes myocardial infarction (MI), a primary subtype of cardiovascular disease (CVD), necrosis and myocardial damage occur due to the blockage of a coronary artery, and cardiac troponins are released into the bloodstream [1]. Among the cardiac troponins (I, T, and C) and other biomarker proteins such as creatine kinase-myocardial band and myoglobin, cardiac troponin I (cTnI) has been considered a gold-standard biomarker for diagnosing MI because of its high clinical sensitivity and cardiac specificity [2].

According to clinical guidelines, cardiac-biomarker testing is an essential prerequisite for MI diagnosis with clinical evaluations based on patient symptoms and history, electrocardiography, and imaging [1,2,3]. With the development of cTnI assays, the specifications of high-sensitivity standard analyzers for cTnI testing have achieved clinically acceptable levels; i.e., a coefficient of variation (CV) of ≤10% at and below the 99th percentile of the upper reference limit for the normal population [4]. However, despite the time-critical nature of MI and recent increases of CVD-related mortality in developing countries, most MI diagnoses based on high-end analyzers operating within clinical guidelines that are available at clinical laboratories require multiple decision processes and a specialized operator, which not only prevents rapid and early diagnosis but also leads to diagnostic inequality [5,6]. Thus, developing a simple, rapid, reliable, and highly sensitive point-of-care test (POCT) for cTnI is urgently needed to alleviate the high rate of mortality and disability caused by MI.

In recent decades, lateral flow assays (LFAs) have demonstrated practical clinical utility as a feasible POCT platform by satisfying most of the World Health Organization’s ASSURED criteria (affordable, sensitive, specific, user-friendly, rapid/robust, equipment-free, and deliverable to end-users) with many real-world products (i.e., home pregnancy tests and rapid diagnostic tests for influenza and malaria) [7,8,9]. Typically, an LFA test strip consists of a relatively simple laminated paper-based structure—i.e., a sample pad, a conjugate pad, a nitrocellulose (NC) membrane, and an absorbent pad—which facilitates mass production [10]. Due to the porous structure of the paper-based materials, the samples migrate through the LFA test strip by passive capillary force without any additional equipment needed for fluid transport. Target recognition initially occurs upon contact with the detection of antibody-labeled 30–40 nm gold nanoparticles (AuNP-Ab conjugates) during the penetration of the conjugate pad and migration through the NC membrane. Capture Abs then recognize the target–conjugate complexes at the test line of the NC membrane, resulting in AuNP-Ab accumulation and the generation of a red-colored visual response with an intensity that depends on the target concentration. However, conventional LFAs employing colloidal AuNPs as a detection label are fundamentally limited in terms of sensitivity and quantitative analysis due to the lower colorimetric response of AuNPs in the presence of low analyte concentrations [11].

To resolve this issue, many studies have employed sensitive labels [11,12,13,14,15,16,17]—i.e., catalytic labels, fluorescence dyes, magnetic particles, quantum dots, upconverting nanoparticles, and surface-enhanced Raman-scattering particles—and have enhanced the analytical sensitivity and quantitative capacity of LFAs. Among these approaches, enzyme-catalyzed LFAs paired with chemiluminescence (CL) detection, which involves the emission of a light signal when the excited intermediate chemicals return to the ground state, is considered to be a promising transduction modality for high-performance LFAs in that the CL method offers (i) no need for incident excitation light, (ii) a higher signal/noise ratio, (iii) and a more extensive dynamic range [11,18,19]. The sensitivities of CL-based LFAs are mainly determined by the design of the conjugate that contains the Ab and enzyme (i.e., horseradish peroxidase (HRP)) because the CL signal that represents the analyte concentration is generated in proportion to the amount of enzyme involved in the analyte recognition. However, most conjugation methods are based on a direct covalent coupling between an Ab and enzyme or co-adsorption and competitive-adsorption of the Ab and enzyme onto the surface of nanomaterials, which limits the enzyme-loading efficiency of every single conjugate [20,21,22].

**Figure 1:**
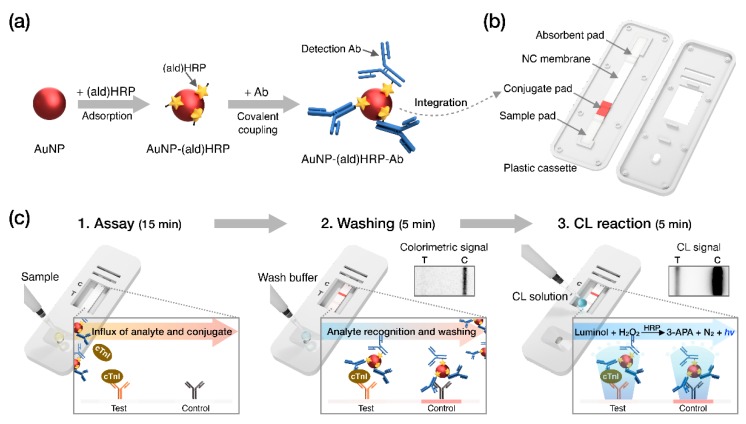
Schematic overview of this study. (**a**) Procedure used to prepare the gold nanoparticle–aldehyde-activated horseradish peroxidase–Ab (AuNP-(ald)HRP-Ab) conjugate. (**b**) Diagram of the lateral flow assay (LFA) test strip. (**c**) Operational process and reactions during the indicated steps. “*hv*” indicates light generated from chemiluminescence (CL) reaction.

Recently, we employed aldehyde-activated HRP, (ald)HRP, to design a new and straightforward conjugation scheme that enables HRP and Ab co-immobilization on the AuNP surface to form an AuNP-(ald)HRP-Ab conjugate. We also demonstrated its enhanced analytical performance over conventional conjugates (Ab-HRP conjugate; synthesized by covalent coupling and AuNP-Ab-HRP conjugate; synthesized by sequential adsorption of Ab and HRP) through CL-based enzyme-linked immunosorbent assays (ELISA) [23]. In this study, we integrated the AuNP-(ald)HRP-Ab conjugate with the LFA biosensing platform for highly sensitive CL-based biomarker testing paired with an HRP-catalyzed luminol-H_2_O_2_ reaction system (Figure 1). The AuNP-(ald)HRP-Ab conjugate was prepared based on the adsorption of (ald)HRP to 15 nm or 40 nm AuNP surfaces and the subsequent covalent coupling between primary amine groups in the Ab and (ald)HRP (Figure 1a). Then, LFA test strips used for the immunoassays were prepared by drying the AuNP-(ald)HRP-Ab conjugate on the conjugate pad (Figure 1b). To evaluate the effect of the AuNP-(ald)HRP-Ab conjugate for enhanced performance in the LFA platform, we compared the analytical sensitivity of the CL response of the AuNP-(ald)HRP-Ab conjugate with that of the colorimetric-based AuNP-Ab conjugate. Moreover, using the CL-based LFA, we tested various concentrations of human cTnI in standard and clinical serum samples to demonstrate its practical and clinical utility over the colorimetric modality of conventional LFAs (Figure 1c).

## 2. Materials and Methods

### 2.1. Preparation and Characterization of AuNP-(ald)HRP-Ab Conjugate Molecules

AuNP conjugates were prepared using 15 nm AuNPs (1×, 1.4 × 10^12^ particles mL^−1^; BBI Solutions, Cardiff, UK) or 40 nm AuNPs (1×, 9.0 × 10^10^ particles mL^−1^; BBI solutions). HRP-conjugated mouse anti-cTnI Ab (4T21C-19C7) was purchased from Hytest (Turku, Finland). The conjugate-synthesis procedures of AuNP-Ab-HRP and AuNP-(ald)HRP-Ab were described in our previous study [23]. Briefly, the AuNP-(ald)HRP-Ab conjugate (ald)HRP (Thermo Fisher Scientific, Waltham, MA, USA) was physically adsorbed to the AuNP surface, and the unbound (ald)HRP particles were eliminated via centrifugal washing. Then, an anti-cTnI Ab (4T21-19C7; Hytest) was linked to the AuNP-(ald)HRP complex through covalent coupling. For the centrifugal washing, we used centrifugation speeds of 21,000× *g* (for 15 nm AuNPs) and 7600× *g* (for 40 nm AuNPs). The AuNP-(ald)HRP-Ab conjugates were concentrated 20× in 50 μL of storage buffer containing 10 mM Fe-EDTA (Sigma), 5% (w/w) trehalose (Sigma), and 0.05% (w/w) BSA (Fitzgerald, Acton, MA, USA) in 10 mM phosphate-buffered saline (PBS; pH 7.4).

The absorbance spectra of AuNPs and AuNP-(ald)HRP-Ab conjugates were measured using a UV-2450 UV-Vis spectrophotometer (Shimadzu, Kyoto, Japan). The hydrodynamic diameter was analyzed by dynamic light scattering (DLS, ELSZ-1000; Otsuka Electronics Co., Ltd., Osaka, Japan).

### 2.2. Optimization of CL Reactions on NC Membranes

Optimization of the concentrations of chemical reagents (luminol, *p*-coumaric acid, 4-iodophenol, and H_2_O_2_) for the CL reactions was performed on test strips composed of an NC membrane and an absorbent pad. The stock solutions were prepared as described previously [23]. The test spots were treated with 1 μL of a 15 nm AuNP-(ald)HRP-Ab conjugate solution (0.1×) and dried for 15 min at 37 °C to physically immobilized the conjugate. To test the enhancer concentration, we used 2 mM luminol and 1 mM H_2_O_2_. To test the luminol concentration, we used 0.5 mM *p*-coumaric acid and 5 mM 4-iodophenol. To optimize the H_2_O_2_ concentration, we used 1 mM luminol and 5 mM 4-iodophenol. The CL intensity was measured after loading 20 μL of the CL reagent solution, prepared in Tris-HCl buffer (100 mM, pH 8.5), onto the NC membrane. After 5 min, the CL response was imaged in high-sensitivity mode (exposure time, 120 s) with a ChemiDoc MP imaging system (Bio-Rad, Hercules, CA, USA). The signal intensity was analyzed using Image Lab software (Bio-Rad). The results were compared by dividing the ratio of CL signal intensity for each test spot by the corresponding background intensity.

### 2.3. Evaluation of the Conjugate Sensitivity

To evaluate the conjugate sensitivity, 1× conjugate solution was serially diluted in the storage buffer. Then, 1 μL of the conjugate at each concentration was spotted onto an NC membrane, and each membrane was dried for 15 min at 37 °C. The color response of the AuNP-Ab conjugate on the NC membrane was imaged with a ChemiDoc MP system in colorimetric mode (exposure time, 0.12 s), after wetting the NC membrane with 20 μL of 1× PBS. The CL response of the AuNP-(ald)HRP-Ab conjugate on the NC membrane was imaged with a ChemiDoc MP system in high-sensitivity mode (exposure time, 300 s), after loading 20 μL of the CL reagent solution. The signal intensity was analyzed using Image Lab software.

### 2.4. Preparation of LFA Test Strips

The LFA test strips used for the immunoreactions consisted of a sample pad (grade 8964; Boreda Biotech, Gyeonggi-do, South Korea), a conjugate pad (grade 6613; Boreda Biotech), an NC membrane (Hi-Flow^TM^ Plus 180, Merck Millipore, Darmstadt, Germany), and an absorbent pad (grade 222; Boreda Biotech). The LFA test strips were prepared via a process involving lateral stacking and lamination of the materials onto a plastic-backed card (PJEAGO, Seoul, South Korea). Individual test strips were produced by cutting assembled test-strip cards with a programmable cutter (TBC-50; cuTex, Gyeonggi-do, South Korea). The procedure used to pre-treat sample pads and conjugate pads was described previously [11]. The conjugate pad was prepared by adding 4× conjugate in storage buffer and drying the pad at 37 °C for 15 min. Test and control lines on the NC membrane were generated using a precision line dispenser (DCI-100; Zeta Corporation, Gyeonggi-do, South Korea) programmed to dispense 1 μg of anti-cTnI capture Abs (4T21-560; Hytest) and 0.2 μg of anti-mouse IgG Ab (Sigma) to the control line.

### 2.5. cTnI Standard and Clinical Sample Testing

To measure cTnI standards and clinical samples, 100 μL of sample solution was injected into the sample inlet of the plastic cassette. To minimize the matrix effect, each serum sample (10 μL) was diluted using assay buffer (90 μL) containing 1% (v/v) Triton X-100 (Sigma) in PBS (10 mM, pH 7.4). After 15 min, washing buffer (1× PBS, 50 μL) was injected into the sample inlet, and each sample was washed for 5 min. Colorimetric images were captured after the washing step. Subsequently, CL reagent solution (20 μL) was loaded on the NC membrane, and each test strip was imaged in high-sensitivity mode (exposure time, 300 s) of the ChemiDoc MP.

Various concentrations of standard cTnI samples were prepared by serially diluting the cTnI antigen (8T62, I-T-C complex; Hytest) in human serum (cTnI free, Hytest). Clinical serum samples containing cTnI (n = 10) were obtained from Chonnam National University Hwasun Hospital (CNUHH). All subjects gave their informed consent for inclusion before they participated in the study. The study was conducted in accordance with the Declaration of Helsinki, and the protocol was approved by the Ethics Committee of CNUHH (institutional review board approval number CNUHH-2019-016). The clinical samples were measured using the Dimension Vista system (Siemens Healthcare, Germany) immediately after collection. Standard cTnI antigen and clinical samples were stored at −80 °C until use.

### 2.6. Statistical Analysis

All experimental data are presented as the mean of three measurements ± the standard deviation (SD). The CV (%) was determined by dividing the SD by the mean (%). The limit of blank (LoB) and limit of detection (LoD) were calculated as follows, based on the guidelines stipulated by the Clinical and Laboratory Standard Institute: LoB = mean_blank_ +1.645 × SD_blank_ and LoD = LoB + 1.645 × SD_low concentration of analyte_. The calibration curve obtained from the cTnI standard sample test was fitted using the Hill1 function of Origin 2018 software (Northampton, MA, USA). Least-squares fitting (log scale) was used to analyze the cTnI test results with clinical samples. Pearson’s correlation coefficient was used for the correlation study.

## 3. Results and Discussion

### 3.1. Characterization of the Conjugates

**Figure 2:**
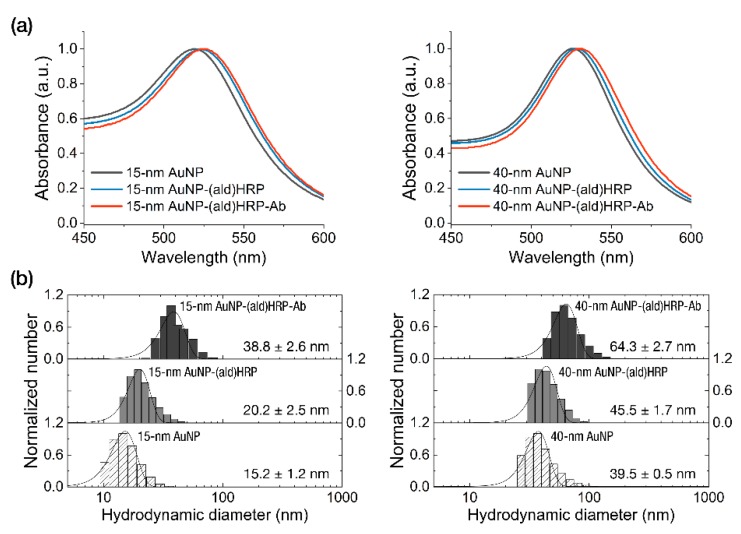
Characterization of the AuNP conjugates. (**a**) Analysis of the absorbance peak shift. (**b**) Analysis of the hydrodynamic diameters after each conjugate step.

The characterization of the conjugates was performed by analyzing the peak shift of absorbance spectra of AuNPs and AuNP-(ald)HRP-Ab conjugates. As shown in Figure 2a, we observed continuous red-shifted absorption peaks at each conjugation step (520 nm → 524 nm → 525.5 nm for the 15 nm AuNP conjugates and 526 nm → 528.5 nm → 530 nm for the 40 nm AuNP conjugates), which indicates successful conjugation.

Using the covalent coupling modality in bioconjugation might cause excessive growth of the conjugate due to the dendronization or physicochemical aggregation, which could potentially lead to multiple biomolecules (i.e., enzyme and Ab) stacking and blocking the bioreceptor’s active/binding sites. Notably, the volume of conjugates intended for use in paper-based porous materials (i.e., NC membrane) is a critical factor for assay efficiency, as the resistance to the conjugate transport through the porous membrane material increases as the conjugates become larger. The low conjugate mobility in LFAs might result in increased background noise (due to conjugate trapping in the membrane fiber matrix), as well as a decreased test line signal, which would directly affect the assay sensitivity.

To investigate whether an excessive growth of conjugate occurs during the conjugate-synthesis based on the covalent coupling of Ab using (ald)HRP, we monitored the size of the AuNP-(ald)HRP-Ab conjugates at each step using DLS measurement. As shown in Figure 2b, we observed a diameter increase of 5–6 nm (on average) after the adsorption of (ald)HRP to the AuNP surface and a sequential diameter increase of 18.6–18.8 nm (on average) after the covalent coupling of antibodies to AuNP-(ald)HRP conjugates, regardless of the size of AuNP. These diameter increases respectively correspond to the size of 1–2 molecules of HRP and antibody (Ab). Moreover, when compared with the average diameter of conventional 40 nm AuNP-Ab conjugate (58.7 ± 2.4 nm) synthesized through physical adsorption, the sizes of our conjugate revealed a smaller (38.8 ± 2.6 nm, 15 nm AuNP-(ald)HRP-Ab) or comparable (64.3 ± 2.7 nm, 40 nm AuNP-(ald)HRP-Ab) level in the range of the nanoscopic dimension (<100 nm), allowing for high conjugate mobility in the porous NC membrane fiber network.

### 3.2. Optimization of the CL Reagents for Membrane Reactions

In contrast to the CL reactions of ELISA, which occur in a stationary, confined, and transparent solution matrix, the CL reactions of LFAs occur within a reagent solution that flows continuously through an opaque NC membrane. To obtain the most intensive CL response in the LFA framework, it is necessary to optimize the concentrations of the reagents used for the HRP-catalyzed luminol-H_2_O_2_ CL reaction system.

**Figure 3:**
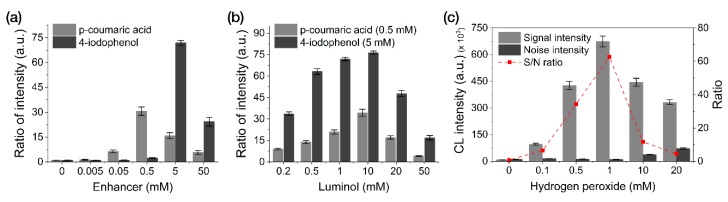
Optimization of the reagent concentrations used for the CL reactions in our LFA framework. (**a**) Enhancers. (**b**) Luminol. (**c**) H_2_O_2_. All data shown are presented as the mean ± SD (n = 3).

We performed an optimization study by modulating the concentrations of enhancers (*p*-coumaric acid and 4-iodophenol), luminol, and H_2_O_2_ with 0.1 M Tris-HCl (pH 8.5) used as the reaction buffer (Figure 3). Enhancer-optimization experiments revealed the most intensive CL response when using 5 mM 4-iodophenol (along with 2 mM luminol and 1 mM H_2_O_2_), followed by 0.5 mM *p*-coumaric acid (Figure 3a). As shown in Figure 3b,c, using 10 mM luminol with 5 mM 4-iodophenol and 1 mM H_2_O_2_ resulted in the highest CL intensity.

Compared to the CL-reagent solution conditions (1 mM 4-iodophenol, 2 mM luminol, and 1 mM H_2_O_2_) optimized for solution-based CL-ELISA reactions in a previous study [23], the CL reagent conditions (5 mM 4-iodophenol, 10 mM luminol, and 1 mM H_2_O_2_) optimized for membrane-based reactions required five-fold greater reagent concentrations (except for H_2_O_2_). We attribute this result to the continuous fluid flow in the LFA, which decreases the probability of the substrate reacting with HRP (per unit time), thus requiring a higher reagent concentration to increase the efficiency of the CL reaction using the LFA framework.

### 3.3. Evaluation of the Conjugate Sensitivity

**Figure 4:**
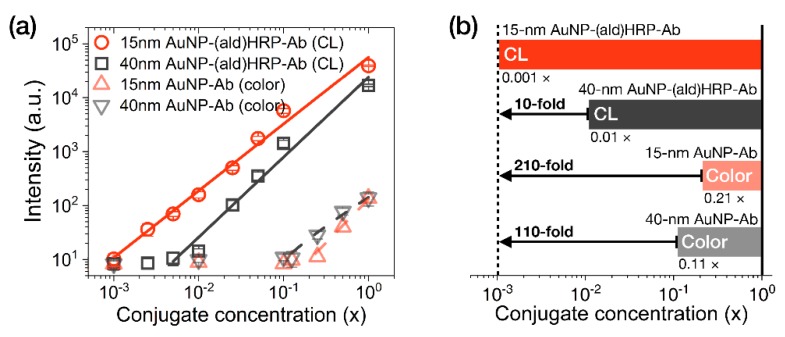
Evaluation of the analytical sensitivity of AuNP conjugates on LFA test strips. (**a**) Intensity plot showing the colorimetric responses of AuNP-Ab conjugates and the CL responses of AuNP-(ald)HRP-Ab conjugates (red line, R^2^ = 0.989; black line, R^2^ = 0.978; gray dashed line, R^2^ = 0.988; red dashed line, R^2^ = 0.954). (**b**) Comparison of the analytical limits of detection (LoDs) of each AuNP conjugate. The numbers labeled below each bar indicate the LoD of the respective conjugates. All conjugates were physically immobilized to the nitrocellulose (NC) membrane through spotting and drying. All data shown in (**a**) represent the mean ± SD (n = 3).

The analytical sensitivity of the AuNP conjugates was investigated by comparing their colorimetric or luminometric signal responses on LFA test strips. AuNP-Ab conjugates were used to evaluate their colorimetric sensitivity, as a representative index of the colorimetric modality of conventional LFAs. AuNP-(ald)HRP-Ab conjugates were used to evaluate the HRP-catalyzed CL modality. Colorimetric and CL responses of serially diluted AuNP conjugates (physically adsorbed and immobilized to NC membranes) are compared in Figure 4.

In contrast to the colorimetric responses observed over an approximately 1-log_10_ range with the AuNP-Ab conjugates (LoD 0.21 × for the 15 nm AuNP-Ab and LoD 0.11× for the 40 nm AuNP-Ab), the CL responses of 15 nm AuNP-(ald)HRP-Ab conjugate and 40 nm AuNP-(ald)HRP-Ab conjugate revealed a linear response over a 3-log_10_ range (LoD 0.001×) and approximately a 2-log_10_ range (LoD 0.01×), respectively (Figure 4a). In particular, the CL response of the 15 nm AuNP-(ald)HRP-Ab conjugate resulted in 10-fold higher analytical sensitivity compared to that of the 40 nm AuNP-(ald)HRP-Ab conjugate and a 110-fold higher colorimetric response when compared with the conventional 40 nm AuNP-Ab conjugate (Figure 4b). The better sensitivity observed with the 15 nm AuNPs could be mainly due to the number of 15 nm AuNPs per unit concentration (1×, 1.4 × 10^12^ particles mL^−1^), which is approximately 15-fold higher than that of the 40 nm AuNPs (1×, 9.0 × 10^10^ particles mL^−1^), as well as the approximately 2.2-fold higher total surface area of 15 nm AuNPs versus the 40 nm AuNPs, resulting in more HRP molecules being conjugated to the AuNPs. Theoretically, this result suggests that using a 15 nm AuNP-(ald)HRP-Ab conjugate for CL-based LFAs will enable the improvement of the analytical sensitivity of the LFA platform by up to 110-fold compared with using a 40 nm AuNP-Ab conjugate for the colorimetric modality. In such a case, the typical LoD of 0.5 to 1 ng·mL^−1^ could potentially be lowered to enable a trace-level analysis of biomarkers at the pg·mL^−1^ level.

### 3.4. cTnI Standard and Clinical Sample Testing

To evaluate the analytical sensitivity of AuNP-(ald)HRP-Ab conjugates in CL-based LFAs, we tested standard cTnI spiked in human serum samples using LFA test strips containing one of three types of AuNP conjugates in the conjugate pad: (i) 40 nm AuNP-Ab (conventional method), (ii) 40 nm AuNP-(ald)HRP-Ab, or (iii) 15 nm AuNP-(ald)HRP-Ab. Moreover, we employed (iv) 15 nm AuNP-Ab-HRP synthesized from the physical adsorption of Ab and HRP and (v) Ab-HRP, which covalently/directly conjugated the HRP to Ab, to validate the effect of the (ald)HRP-based conjugation methods compared to conventional HRP-containing conjugates. The total testing time was 25 min, which involved three sequential steps, namely the immunoassay (15 min), the test-strip washing step (5 min), and the CL reaction and signal accumulation step (5 min).

**Figure 5:**
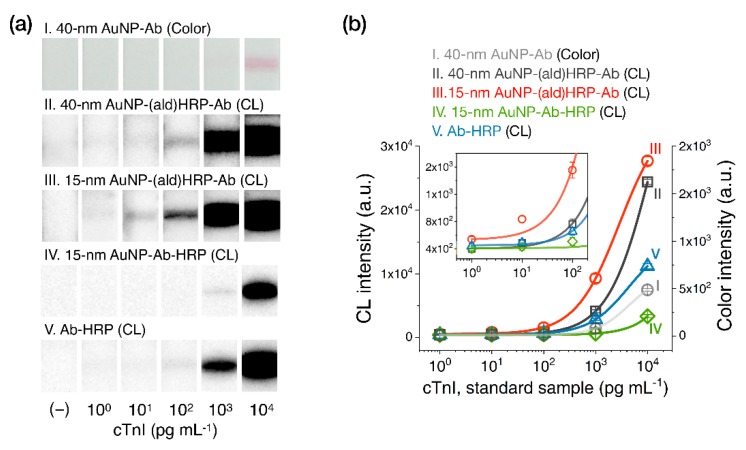
Results of cardiac troponin I (cTnI) standard sample testing with the LFA. (**a**) Test line images. (**b**) Test line intensity plot. All data shown in (**b**) represent the mean ± SD (n = 3). The cTnI levels shown reflect the final concentrations used in the assays.

Figure 5a shows representative test-line images (colorimetric and CL responses) of LFA test strips when testing cTnI standards, and Figure 5b displays the corresponding signal intensities. Using the AuNP-(ald)HRP-Ab conjugates, the test-line signal revealed concentration-dependent CL responses over a 4-log_10_ range (10^0^–10^4^·pg·mL^−1^) for the 15 nm AuNP-(ald)HRP-Ab conjugate (LoD = 5.6 pg·mL^−1^; CV = 2.3–8.4% for all measurements) or a 3-log_10_ range (10^1^–10^4^ pg·mL^−1^) for the 40 nm AuNP-(ald)HRP-Ab conjugate (LoD = 30 pg·mL^−1^; CV = 1.5–9.5% for all measurements). Compared to the conventional colorimetric responses obtained using the 40 nm AuNP-Ab conjugate, with a detection range of ~1 log_10_ range (10^3^–10^4^ pg·mL^−1^) and an LoD of 607 pg·mL^−1^, the 15 nm AuNP-(ald)HRP-Ab conjugate had an approximately 81-fold higher sensitivity. Considering the clinical guideline for cTnI assays that suggests cTnI measurements at or below the 99th percentile upper reference limit of the healthy population (potentially, 8–58 pg·mL^−1^) with a CV of 10% [24], the standard cTnI testing results using the 15 nm AuNP-(ald)HRP-Ab conjugate were within the clinically acceptable level and demonstrated the potential utility of CL-based LFAs for MI diagnosis.

Furthermore, the result of the 15 nm AuNP-(ald)HRP-Ab conjugate revealed an enhanced response compared with that of the 15 nm AuNP-Ab-HRP (LoD = 417 pg·mL^−1^; CV = 5.3–11.4% for all measurements) and Ab-HRP (LoD = 72 pg·mL^−1^; CV = 3.7–9.4% for all measurements) conjugates. These results further support the CL-based ELISA results described in our previous study [23], demonstrating that our conjugation method based on the adsorption of (ald)HRP and covalent coupling of Ab could improve the assay performance of LFAs compared to the conventional competitive adsorption of HRP and Ab to the AuNP surface, as well as the direct covalent coupling of HRP to Ab.

**Figure 6:**
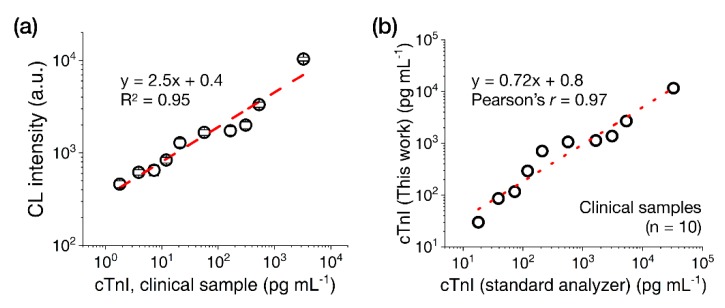
Results of cTnI clinical sample testing with the LFA. (**a**) Test results obtained with cTnI clinical serum samples (n = 10). (**b**) Correlation between the measured cTnI concentration in clinical samples found using the 15 nm AuNP-(ald)HRP-Ab conjugate and standard analyzer. All data shown in (**a**) represent the mean ± SD (n = 3). The cTnI levels indicated in **(a)** reflect the final concentrations used in the assays. The cTnI levels indicated in (**b**) represent the initial concentrations of the samples.

Next, we tested cTnI clinical serum samples (n = 10, 18–32,700 pg·mL^−1^) with the CL-based LFA platform using the 15 nm AuNP-(ald)HRP-Ab conjugate. As shown in Figure 6a, the clinical samples showed concentration-dependent increases of CL signal intensities with a CV range of 4.8%–10.4%. Although the cTnI values calculated using the LFA were consistently lower than those measured using the standard analyzer (which may have originated from the systemic differences that normally occur between cTnI assays) [25], comparing the values obtained with both assays revealed a high correlation (Pearson’s r = 0.97; Figure 6b), demonstrating the utility of the CL-based LFA platform with 15 nm AuNP-(ald)HRP-Ab conjugate for measuring cTnI levels in clinical samples.

## 4. Conclusions

In this study, we demonstrated the applicability of AuNP-(ald)HRP-Ab conjugates for the highly sensitive detection of cTnI proteins with a CL-based LFA biosensing platform. The synthesis of the conjugate based on the physical adsorption of (ald)HRP to the AuNP surface and subsequent covalent coupling of Ab to (ald)HRP resulted in a nanoscopic dimension of the conjugates, which is suitable for high mobility on the porous membrane network of LFAs. Compared to the analytical sensitivity of the conventionally labeled 40 nm AuNP-Ab conjugate, which has been used as a colorimetric readout modality in LFAs, the 15 nm AuNP-(ald)HRP-Ab conjugate revealed highly enhanced sensitivity (110-fold) when paired with an HRP-catalyzed luminol-H_2_O_2_ CL reaction system. Using this strategy, we measured cTnI levels in standard serum samples in 25 min, resulting in high sensitivity (LoD of 5.6 pg·mL^−1^) and reliable precision (with a CV of 2.3%–8.4%), which are acceptable based on clinical guidelines. Furthermore, measuring the cTnI levels in clinical serum samples revealed a high correlation (Pearson’s r = 0.97) with standard analyzers, indicating its potential utility in cTnI testing for MI diagnosis.

Currently, most of the cTnI testing for MI diagnosis is performed in central clinical laboratories within hospitals, where multiple decision steps and a high workload exist, thereby ironically delaying the diagnosis of MI, which requires time-critical diagnosis and clinical action. Given this background, we expect that the use of the CL-based LFA proposed in this study will enable rapid MI and cTnI monitoring diagnoses through the patient-centered POCT at the emergency department, clinic, or rural healthcare center. In particular, the highly sensitive CL-based sensing modality may facilitate the high-sensitivity detection of early-stage MI patients with cTnI adjacent to the clinical cut-off level. However, prior to clinical implementation, a more systemic optimization of the testing results is required to match contemporary standard analyzers for more precise and reliable testing. Moreover, a stability study on maintaining the activity of HRP and CL reagents is essential for the application to various resource setting levels. With these developments, we foresee that our CL-based LFA could be applied to facilitate MI diagnosis in point-of-care contexts.
